# Functional Connectivity Analysis in Motor-Imagery Brain Computer Interfaces

**DOI:** 10.3389/fnhum.2021.732946

**Published:** 2021-10-15

**Authors:** Nikki Leeuwis, Sue Yoon, Maryam Alimardani

**Affiliations:** Department of Cognitive Science and Artificial Intelligence, Tilburg University, Tilburg, Netherlands

**Keywords:** motor imagery (MI), brain computer interface (BCI), BCI inefficiency, electroencephalography (EEG), functional connectivity (FC), phase synchronization

## Abstract

Motor Imagery BCI systems have a high rate of users that are not capable of modulating their brain activity accurately enough to communicate with the system. Several studies have identified psychological, cognitive, and neurophysiological measures that might explain this MI-BCI inefficiency. Traditional research had focused on mu suppression in the sensorimotor area in order to classify imagery, but this does not reflect the true dynamics that underlie motor imagery. Functional connectivity reflects the interaction between brain regions during the MI task and resting-state network and is a promising tool in improving MI-BCI classification. In this study, 54 novice MI-BCI users were split into two groups based on their accuracy and their functional connectivity was compared in three network scales (Global, Large and Local scale) during the resting-state, left vs. right-hand motor imagery task, and the transition between the two phases. Our comparison of High and Low BCI performers showed that in the alpha band, functional connectivity in the right hemisphere was increased in High compared to Low aptitude MI-BCI users during motor imagery. These findings contribute to the existing literature that indeed connectivity might be a valuable feature in MI-BCI classification and in solving the MI-BCI inefficiency problem.

## Introduction

Brain-Computer Interface (BCI) is a system that outputs an action based on the classification of the user’s brain waves. The technique enables humans to interact with the physical environment and external devices without having to move muscles (Wolpaw et al., 2002). This is a solution for disabled bodies—for example, caused by a stroke (Wolpaw et al., 2002), who can thereby control an exoskeleton (Jeong et al., 2020), robot arm (Edelman et al., 2019), or wheelchair (Kim et al., 2016).

The brain signals as input for the BCI are most commonly measured with electroencephalography (EEG) because it is non-invasive, low-cost, and user-friendly compared to other imaging techniques. There are various BCI paradigms that employ different tasks and EEG components for operation (e.g., P300, SSVEP, etc. see Abiri et al., 2019 for a review). Motor imagery BCI (MI-BCI) systems rely on the mental execution of a movement, which changes brain activity in the motor cortex (Pfurtscheller and Neuper, 2001). The system classifies these changes and thereby sends a command to the external device (Wolpaw et al., 2002). MI-BCIs have the advantage of not requiring external stimuli (as opposed to reactive BCIs) however they require extensive training until the user is capable of producing ideal brain activity patterns for the system to classify (Wolpaw et al., 2002).

However, Allison and Neuper (2010) concluded that 15–30 percent of users are incapable of using a MI-BCI system even after training. This lack of control is traditionally called “BCI illiteracy” (Allison and Neuper, 2010), which is more recently replaced with the term “BCI inefficiency” to stress the fact that the users are not solely responsible for inaccurate classification (Thompson, 2019); the issue of BCI inefficiency arises with the variations in brain signals between different subjects and experiments (Lotte et al., 2007; Lee et al., 2019a) and its prevalence has been investigated by multiple studies as well (Jeunet et al., 2016; Lee et al., 2019a; Meng and He, 2019).

The variability in BCI performance has been related to a variety of cognitive, psychological, and neurophysiological factors (e.g., Jeunet et al., 2015; Leeuwis et al., 2021a) which gives rise to the intra- and intersubject variability in EEG brain activity patterns (Saha and Baumert, 2020). Plenty of studies have investigated user’s traits, psychological states, and cognitive abilities to relate them to BCI performance (e.g., Jeunet et al., 2015; Leeuwis et al., 2021a), and while some variability is explained, the holy grail has yet to be found. Identifying inefficient users and the underlying mechanisms of BCI inefficiency is important as it will allow researchers to: (1) select suitable subjects for their experiments; (2) adapt the training strategy and duration to each user; and (3) report subject-dependent results that would make the comparison between studies easier (Sannelli et al., 2019).

Research into the neurophysiological factors has mainly focused on pattern changes in the sensorimotor rhythm (SMR; Blankertz et al., 2010) known as mu suppression (Wolpaw and McFarland, 2004). Mu suppression refers to Event-Related (De)Synchronization (ERD/ERS) within alpha/mu band (8–13 Hz; Penaloza et al., 2018) and is seen in both motor execution (Duann and Chiou, 2016) and motor imagery (Pfurtscheller and Neuper, 2001) and therefore is widely applied in MI-BCI systems (e.g., Pfurtscheller et al., 2006; Blankertz et al., 2008). For instance, BCI inefficiency is usually attributed to a lower SMR amplitude during resting-state (Zhang et al., 2020) and MI task (Shu et al., 2018), implicating that the MI-induced SMR-modulation is smaller, which results in insufficient discrimination of brain activity patterns for the system to correctly translate the users’ intentions (Zhang et al., 2020).

While the SMR activity can be enhanced by conducting multiple training sessions, the inter-subject variability is still present after training and thereby contributes to user inefficiency (Saha and Baumert, 2020). This variability arises as the frequency bands and cortical regions in which MI-related activations appear are not consistent for all subjects, which leads to the unreliability of ERD/ERS analysis for different subjects (Hamedi et al., 2016; Benaroch et al., 2021). Focusing on one specific region while neglecting its interactions with other regions, oversimplifies the real phenomenon of motor imagery; the system’s collective behavior should be understood to fully capture the brain activity during motor imagery (Gonzalez-Astudillo et al., 2020). Therefore, especially the inefficient BCI users with low resting-state SMR might benefit from new measures; such as brain connectivity analysis (Hamedi et al., 2016; Zhang et al., 2020).

Brain connectivity analysis provides a tool to inspect the interaction between brain regions during the MI task; it quantifies the exchange of information and its relevance to the user’s BCI performance (Wang et al., 2006; Zhang et al., 2017). This is done with connectivity measures such as functional connectivity and effective connectivity (Hamedi et al., 2016). Functional connectivity is explained as statistical dependencies between brain regions, and effective connectivity gives directionality to this exchange of information (Lee et al., 2019b). Previous reports suggest that inefficient BCI users exhibit different brain connectivity at the baseline resting-state, and this can be used to predict their performance during the task (i.e., Zhang et al., 2015; Lee et al., 2020). For instance, Zhang et al. (2015) reported that the average functional connectivity at resting-state is positively correlated with BCI performance, indicating that a higher EEG connectivity during resting-state was related to better BCI accuracies. In addition, Lee et al. (2020) observed significantly higher effective connectivity from the supplementary motor area (SMA) to the right dorsolateral prefrontal cortex (DLPFC) in high aptitude BCI users during resting-state when compared to low aptitude performers. Implementing these findings, research showed that functional connectivity during the MI task (Yi et al., 2014; Stefano Filho et al., 2017; Gu et al., 2020; Vidaurre et al., 2020; etc.) or the change in functional connectivity from resting-state to MI task can be used as a feature for MI-BCI classification (Gonuguntla et al., 2016; Hamedi et al., 2016). This means that MI-BCI performance cannot be solely dependent on the resting-state EEG and that the user’s ability to reorganize brain activity during the MI task may play a critical role in determining the success of the BCI control.

In the former studies, both effective connectivity (e.g., Lee et al., 2020) and functional connectivity (e.g., Vidaurre et al., 2020) measures have been investigated, however, these studies employed various metrics of connectivity including coherence, phase synchronization, phase-slope index, etc., which employ different algorithms and hence vary in their interpretation (Bastos and Schoffelen, 2016). However, to fully tackle the disadvantages of EEG, such as artifacts and inter-trial/inter-subject amplitude variability, phase-based relationships (e.g., phase synchronization) might provide the best functional connectivity measure of spatially distributed regions that are active during mental task execution (Caicedo-Acosta et al., 2021). Functional connectivity features measured by the phase lag index (PLI) and phase-locking value (PLV) can discriminate between different MI tasks (Stefano Filho et al., 2018; Caicedo-Acosta et al., 2021), and therefore are a promising tool to identify potential non-learners (Caicedo-Acosta et al., 2021).

Besides the variety in applied algorithms, different networks of connectivity are observed in previous literature. Some studies computed connectivity as average synchronization between distributed brain regions i.e., an average of all electrode connections over the scalp (e.g., Zhang et al., 2015). Others make the distinction between local scale and large scale (e.g., Wang et al., 2006; Zhang et al., 2014; Vidaurre et al., 2020). In addition, some researchers worked with source localization algorithms before applying connectivity measures (e.g., Gu et al., 2020; Lee et al., 2020; Vidaurre et al., 2020). Within this distribution of scales, especially separating connectivity values for intra- and inter-hemispheric activity makes sense as the MI task is lateralized between left- and right-hand movements which are represented contralateral in the brain: the ERD on the left side of the motor region (C3) is observed in right-hand MI, whereas that on the right side of the motor region (C4) is observed in left-hand MI (Luo et al., 2021).

While past studies have investigated the potential of brain connectivity analysis in identifying the mechanisms of BCI inefficiency, the literature remains inconclusive regarding the role of connectivity measures as these studies employed a variety of connectivity algorithms, on a variety of scalp locations and in a variety of tasks. Most studies showing the value of connectivity have been working on small datasets, comparing results at an individual level instead of a statistically meaningful group level. Therefore, it is important to validate the efficacy of brain connectivity as a potential predictor of MI-BCI performance by using a larger dataset that represents the large inter-subject variability that exists among BCI users (Leeuwis and Alimardani, 2020).

In this study, we aim to examine the relationship between EEG connectivity during the MI task and users’ BCI performance. By comparing two groups of High and Low BCI performers in a large dataset (*N* = 54), this study intends to investigate if successful MI task execution (i.e., better BCI performance) is associated with establishing a different (perhaps stronger) connectivity pattern between brain areas.

As phase synchronization is a promising measure in predicting MI-BCI performance, which is also robust to artifact and inter-subject amplitude variability (Caicedo-Acosta et al., 2021), PLV is employed as a measure of functional connectivity in this study.

In addition, this study intends to bring consistency to literature by evaluating EEG connectivity at different network scales. We explored the connectivity difference between High vs. Low aptitude groups in the sensorimotor areas by examining all different scales that are proposed in previous research: the average connectivity over all electrodes (e.g., Zhang et al., 2015), and the distinction between local scale and large scale (e.g., Wang et al., 2006; Zhang et al., 2014; Vidaurre et al., 2020). Since intra-hemispheric connectivity might depend on the directionality of trials, in combination with the dominant hand of the subjects, the directionality of trials was also considered as a factor in the analysis.

Thus, the present study will uncover if the PLV values during left MI trials are significantly different from PLV values during the right trials in either high or low performers on three scales of connectivity: (1) the average connectivity between all connections; (2) large scale connectivity based on connections between the left and right motor areas; and (3) local scale connectivity based in either left or right motor areas. The large number of participants and the incorporating of this number of connectivity scales is unique in the literature and will therefore contribute to a better understanding of functional connectivity and its relation to motor imagery.

## Materials and Methods

### Experiment

#### Participants

The data was collected by Leeuwis et al. (2021b). The dataset comprises 55 novice subjects (*M_Age_* = 20.71, *SD_Age_* = 3.52, 36 females, 19 males). Subjects were all right-handed with (corrected to) normal vision by criteria for participation.

#### EEG Signals

EEG was recorded from 16 electrodes according to the 10–20 international system (F3, Fz, F4, FC1, FC5, FC2, FC6, C3, Cz, C4, CP1, CP5, CP2, CP6, T7, and T8) while the subjects completed a MI-BCI task. The reference was set on the right earlobe and a ground electrode on AFz. EEG signals were amplified by a g.Nautilus amplifier (g.tec Medical Engineering, Austria) at the sampling rate of 250 Hz.

#### Motor Imagery Task and the BCI System

**Figure 1:**
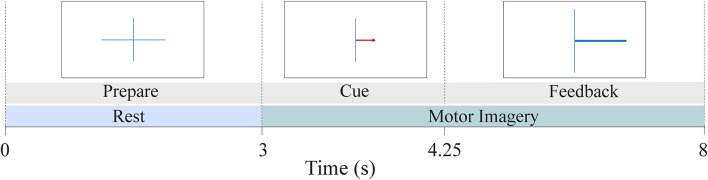
MI-BCI paradigm during the three feedback runs. The dataset included a total of 120 trials (60 right and 60 left-hand trials) per subject. Each trial lasted 8 s. The first 3 s of the trial served as the rest phase while the last 5 s provided motor imagery data.

Details of the experimental procedure can be found in Leeuwis et al. (2021a,b). The BCI paradigm included four runs: it started with one non-feedback calibration run followed by three feedback runs. In this study, we work with the data recorded during the three feedback runs, hence excluding the calibration trials. Each run consisted of 20 left- and 20 right-hand trials, resulting in 40 trials per run. Each trial took 8 s and started with a fixation cross shown for 3 s (Figure 1). A red arrow cued the direction in which the participant had to imagine movement. This arrow was presented for 1.25 s. After the cue, a feedback bar indicated the direction and certainty of the BCI classifier’s prediction to the participant.

The online BCI classifier used g.BSanalyze (g.tec Medical Engineering, Austria), which is a Simulink-based high-speed online processing package. The classifier relied on Common Spatial Patterns (CSP) algorithm to compute ERD/ERS in each trial. The classifier was recalibrated after every run. Thus, the classifier parameters were recomputed based on the latest run. In doing so, the classifier was optimized to the strategy of the user throughout the session.

### Data Analysis

#### Low and High BCI Groups

Subjects were split into two groups of Low and High performers based on their online BCI performance. The accuracies obtained from all trials in the three feedback runs were averaged and a median split was performed. The subject with the median value was excluded from the analysis, which left both High and Low performance groups with 27 subjects. The median score was 71%, which is closely compatible with the inefficiency threshold of 70% in previous studies (Lee et al., 2019a; Meng and He, 2019). The median split provided a balanced group comparison, which in return improved statistical strength. The Low performers group included 11 males and 16 females (*M_Age_* = 21.4, *SD_Age_* = 4.44). The High performers group were 19 females and eight males (*M_Age_* = 20.2, *SD_Age_* = 1.94). Their BCI performance is indicated in Table 1.

**Table 1:** Average BCI performance for high and low BCI performers.

	*M*	*SD*
High aptitude users	77.17	5.44
Low aptitude users	67.19	2.96

#### EEG Pre-Processing and PLV Calculation

The EEG data were re-referenced using common average referencing (CAR) and then band-pass filtered between 8 and 30 Hz using the MNE python package (Gramfort et al., 2013). A Laplacian spatial filter was applied to reduce the effects of volume conduction (Cohen, 2014; Kayser and Tenke, 2015). Following the default settings, the regularization parameter γ was 1e-5 and the stiffness of the spline was 4 (Gramfort et al., 2013; Cohen, 2014). The preprocessed EEG signals were used to extract PLV by using a complex Morlet wavelet (CMW) as a kernel. CMW is defined as


$$\psi (t)\;=\;e^{j2\pi f_{c}t}\;e{\;}^{-t^{2}/f_{b}}\;/\;\sqrt{\pi f_{b}}$$

where *f_b_* is the bandwidth parameter and *f_c_* is the center frequency (He et al., 2011). For alpha frequency analyses, *f_c_* was 10.5 and *f_b_* was 0.3. For beta band frequency analyses, *f_c_* was 21.5 and *f_b_* was 0.13.

Continuous wavelet transform using a CMW enables detecting changes in the frequency domain that occur in different time periods. CMW is popular in the time-frequency decomposition of EEG signals because it uses a Gaussian-modulated sinusoid and therefore its shape resembles the neurophysiological signals (Kopal et al., 2014).

The functional connectivity between different sites of the brain was calculated in terms of phase synchronization. Investigating a synchronization between brain signals collected from different sites can unveil how the cortical regions communicate with each other (Gray et al., 1989). Phase synchronization analysis started with the effort to understand the phase-locking phenomenon in which a constant phase difference between two signals lasts for a short period of time (Rosenblum et al., 1996; Mezeiová and Paluš, 2012). Phase synchronization analysis is conducted by quantifying this phenomenon into a phase-locking value (PLV) that is obtained by the equation below


(1) $$PLV(t)=\;\frac{1}{N}\left|\sum_{n=1}^{N}\;e^{i\theta (t,n)}\right|$$

where *θ* (t,n) stands for the phase difference *ϕ*_1_(*t,n*) − *ϕ*_2_(*t,n*) on time bin *t* for each trial *n* in [1,…,*N*] (Lachaux et al., 1999). Time bin t reflected 3 s for resting state and 3.5 s for MI task with zero overlap. The PLV was calculated for each pair of electrodes in the dataset.

The PLV ranges from 0–1, with 1 indicating complete phase synchronization and 0 indicating no phase synchronization. Complete phase synchronization appears when the two compared EEG signals possess indistinguishably the same characteristics (Mezeiová and Paluš, 2012). By using phase synchronization to quantify functional connectivity, the information carries more refined information than other measures because it focuses on the phase of the signal regardless of the amplitude of the wave which can change due to various artifacts including the body movements.

#### Time Course of Functional Connectivity

Past studies investigated EEG connectivity either during resting-state (e.g., Zhang et al., 2015; Lee et al., 2020) or the motor imagery task (e.g., Gu et al., 2020; Vidaurre et al., 2020) to associate it with BCI inefficiency. This study aimed to provide a comprehensive analysis of EEG functional connectivity by examining the difference between High and Low BCI performance users at rest, during the motor imagery task, and also the change of functional connectivity that took place between the two phases. Therefore, for each trial, PLV was calculated in three ways: (1) PLV during pre-MI rest (PLV_Rest_); (2) PLV during the motor imagery task (PLV_MI_); and (3) the change of PLV from rest to the MI task (ΔPLV = PLV_MI_ − PLV_Rest_). The last measure serves as a quantification of the neuromodulation that is expected to take place when a user switches from rest to MI task. Figure 1 provides an example of the chronological order of the BCI task during each feedback trial. The first 3 s of each trial were marked as rest, followed by the MI task in the next 5 s. Considering the time needed for the user to engage in the task and the impact of feedback toward the end of the trial, only 3.5 s of the MI task (between 1 s and 4.5 s after cue onset) were evaluated for the computation of the PLV-value. This duration of the MI segment was determined by making a consensus between Marchesotti et al. (2016) and Lee et al. (2019a).

#### Localization of Functional Connectivity

**Figure 2:**
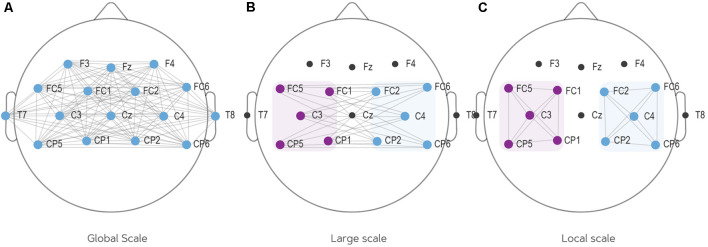
The three scales of connectivity employed in this study. **(A)** Global scale connectivity: computes average PLV of all electrodes. **(B)** Large scale connectivity: computes average PLV from the left to the right hemisphere. **(C)** Local scale connectivity: computes local connectivity on two locations: the left hemisphere (C3 and all surrounding electrodes) and the right hemisphere (C4 and all surrounding electrodes). PLV, phase-locking value.

Different scales of connectivity are observed in previous literature. To create the most complete overview of different scales employed by past studies, the current study employed three different connectivity scales: (1) a global PLV which takes the average of connectivity over all electrodes (e.g., Zhang et al., 2015); (2) a large scale PLV which tests the connectivity between the left hub (FC5, FC1, C3, CP5, CP1) and right hub (FC6, FC2, C4, CP6, CP2); and (3) a local scale PLV which looks at connectivity within either the left or right hub. This distinction between large and local scale connectivity has also been made by past research (e.g., Wang et al., 2006; Zhang et al., 2014; Vidaurre et al., 2020). Large scale connectivity targets the inter-hemispheric network whereas local scale targets intra-hemispheric network. It is plausible for the connectivity during the MI task to be considered in both scales as the task is lateralized between left- and right-hand movements which are contralaterally represented in the brain. Figure 2 illustrates the connections included in every network scale. Note that for the local scale, two separate analyses were conducted: one for the left hemisphere (C3 neighborhood) and one for the right hemisphere (C4 neighborhood).

#### Statistical Analysis

Factorial ANOVAs with permutation were run for each network scale, where the dependent variable of the ANOVA was the average PLV value generated from the given electrode configurations and independent variables were BCI performance groups (High vs. Low) and MI hand (Left vs. Right). The interaction term was included in the model. The number of permutations was 999 for all analyses. When applicable, *post hoc* analyses were conducted with a two-sample *t*-test with a permutation test.

Since several analyses were done, results had to be corrected for multiple comparisons. In each network scale, connectivity values in three activity types (PLV_MI_, PLV_Rest_, ΔPLV) were compared in two frequency bands (alpha, beta). Thus, following the Bonferroni correction, the significance level was adjusted to 0.05/6 = 0.008.

## Results

Subjects were grouped into High and Low aptitude BCI users by splitting the BCI online performance at the median value (71.00%) which created two groups of 27 subjects. The mean and SD of BCI online performance in each group are shown in Table 1. The PLV values for all electrode connections can be found in the Supplementary Material.

The results of factorial ANOVAs within the alpha band for global scale, large scale, and local scale connectivity are shown in Table 2. No significant effects were found for global scale, large scale and local scale left hemisphere connectivity in any of the phases or any of the frequency bands. Only for the right hemisphere local connectivity, the test showed significantly different PLV between the BCI performance groups during the motor imagery phase in the alpha band (*F*_(1,1,1,320)_ = 8.36, *p* = 0.005) although there was no main effect for the MI hand or an interaction effect between the two factors. The results in the beta band were non-significant for all scales.

**Table 2:** Results of the factorial ANOVA for global scale, large scale, and local scale connectivity in the alpha frequency band.

	*PLV_MI_*			*PLV_Rest_*			*ΔPLV*		
	*F*	*p*	*Post hoc*	*F*	*p*	*Post hoc*	*F*	*p*	*Post hoc*
	**Global Scale (Average of all connections)**								
	***Alpha band***								
BCI group	0.311	0.571		0.007	0.932		1.777	0.185	
Right/Left (RL)	0.321	0.592		0.110	0.754		0.256	0.601	
BCI:RL	0.126	0.722		0.009	0.906		0.292	0.564	
	***Beta band***								
BCI group	1.238	0.245		0.898	0.381		0.710	0.420	
Right/left (RL)	0.015	0.884		0.179	0.652		0.708	0.379	
BCI:RL	0.030	0.874		0.017	0.894		0.032	0.869	
	**Large scale (C3 and surrounding electrodes × C4 and surrounding electrodes)**								
	***Alpha band***								
BCI group	2.710	0.101		0.064	0.450		2.589	0.114	
Right/Left (RL)	0.029	0.854		0.000	0.997		0.097	0.781	
BCI:RL	0.092	0.726		0.000	0.980		0.266	0.629	
	***Beta band***								
BCI group	2.122	0.128		1.600	0.209		0.515	0.487	
Right/Left (RL)	0.001	0.997		0.167	0.658		0.838	0.340	
BCI:RL	0.047	0.980		0.030	0.868		0.020	0.886	
	**Local scale (C3 × surrounding electrodes)**								
	***Alpha band***								
BCI group	0.030	0.851		0.015	0.892		0.014	0.922	
Right/Left (RL)	0.045	0.818		0.089	0.775		0.028	0.859	
BCI:RL	0.086	0.759		0.017	0.903		0.898	0.324	
	***Beta band***								
BCI group	1.224	0.267		0.735	0.411		1.114	0.290	
Right/Left (RL)	0.457	0.482		0.110	0.741		1.442	0.223	
BCI:RL	0.040	0.847		0.006	0.940		0.740	0.360	
	**Local scale (C4 × surrounding electrodes)**								
	***Alpha band***								
BCI group	8.358	0.005*	High > low (*p* = 0.002)	5.824	**0.012**		2.577	0.113	
Right/Left (RL)	0.198	0.663		0.003	0.959		0.889	0.366	
BCI:RL	0.503	0.470		0.045	0.845		1.511	0.226	
	***Beta band***								
BCI group	3.486	0.071		2.743	0.094		1.659	0.199	
Right/Left (RL)	0.085	0.764		0.006	0.924		1.979	0.165	
BCI:RL	0.017	0.884		0.001	0.959		0.414	0.530	

In each scale, average PLV during the Rest, MI, and transition of the two phases (ΔPLV) was compared between BCI performance groups (High vs. Low) and MI hand (Right vs. Left). The interaction between the two variables was included in the model. The significance level was adjusted to 0.008 using Bonferroni correction. PLV, phase-locking value. Bold values indicate significant effects before Bonferroni correction and * indicates significant effect after Bonferroni correction which is then followed by a post-hoc analysis.

**Figure 3:**
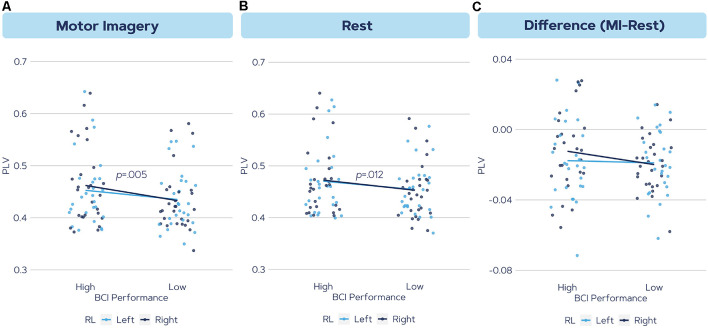
The phase synchronization values in the alpha frequency band in the local scale right hemisphere during **(A)** motor imagery, **(B)** rest, and **(C)** the difference between motor imagery connectivity and rest. The color indicates left and right trials. Results indicated a significant difference in **(A)** and a trend in **(B)**.

*Post hoc* analysis revealed that the high aptitude users had significantly higher PLV values compared to the low aptitude BCI users (*p* = 0.002). Figure 3 demonstrates the alpha band PLV in the local scale right hemisphere for both high and low groups as computed in three ways; during Motor Imagery, Rest, and the change from Rest to MI.

## Discussion

This study aimed to establish the efficacy of EEG functional connectivity in recognizing BCI inefficient users in a motor imagery paradigm. Phase synchronization (PLV) was compared in two groups of High and Low aptitude users, each containing 27 subjects, in different brain network scales (global, large, and local scale) and different timelines of the motor imagery task (Rest, MI, and MI − Rest). Results indicated a significantly higher alpha band connectivity in the right hemisphere (local scale) for the high performing users during the motor imagery task. The implications of these findings are discussed in the following sections.

### The Impact of Connectivity During Motor Imagery

Several studies in the past have identified connectivity as a marker for MI-BCI accuracy. Wang et al. (2006) were early to show that functional connectivity as measured through phase synchronization could serve as a predictor of MI-BCI performance; phase synchrony between supplementary motor area (SMA) and primary motor areas (M1) differed between left and right MI trials. Later, Zhang et al. (2014) calculated connectivity metrics on a two-class MI-BCI and revealed that connectivity within left and right hemispheres differed for both hands during motor imagery. This would enable within-hemisphere connectivity values as a possible feature in MI-BCI classification (Zhang et al., 2014). Subsequently, Gu et al. (2020) used this feature to show that within-hemispheres functional connectivity during the MI task could enhance BCI classification of foot imagery. Although Stefano Filho et al. (2017) found that regular Power Spectral Density (PSD) methods provide slightly better accuracies than functional connectivity as a feature for motor imagery classification, Wang et al. (2019) showed that classification with Common Spatial Patterns (CSP) can be improved by adding synchronization as a feature. Here, synchronization was calculated with cross-correlation and PLV, which were then compared and combined with CSP. Combining CSP with cross-correlation functions improved classification accuracy and performed better than CSP combined with PLV (Wang et al., 2019). Similarly, Zhang et al. (2019) used a fusion of functional connectivity and event-related desynchronization (ERD) features and observed that MI classification accuracy increased such that 4 out of 12 inefficient users performed above the efficiency threshold of 70% accuracy. This result is particularly of interest as it shows that low performing users may engage in the MI task in a different way that can only be captured by brain network features. Our study confirms that a difference in dynamic brain network patterns between High and Low performers exists, especially during motor imagery, although not in all network scales that are proposed in literature.

These results may be discussed in the context of the directionality of connectivity. Baxter et al. (2017) employed an inference approach and showed that connectivity can be altered with transcranial direct current stimulation before performing the BCI task. The increased connectivity was correlated with improved motor imagery performance in both hands when there was a strongly connected input from the (ipsilateral) posterior parietal cortex (PPC) or premotor cortex (PMC) to the sensorimotor cortex (SMC). Performance decreased when the connection was reversed. Their results confirmed that motor imagery-induced connectivity relates to MI-BCI performance, but also suggest that the inflow or outflow across regions may play an important role in determining MI performance. The in- and outflow specifically were not tested in this study as we employed phase-based connectivity analysis, however, the implication of strengthening connections in the motor areas holds. Additionally, this method can be applied to change the cortical excitability of users before the task in order to upregulate the connectivity and thereby promote learning.

### The Impact of Resting-State Connectivity

Following the conclusions of Zhang et al. (2020) that resting-state SMR might be an important identifier of BCI inefficient users and the recent findings of Lee et al. (2020) who showed that effective connectivity during Rest was already a predictor of BCI performance, we hypothesized that PLV during the Rest phase would be greater in High aptitude MI-BCI users compared to Low aptitude users. This hypothesis was in line with the report of Vidaurre et al. (2020) who showed that functional connectivity during both Rest and MI were correlated to online feedback performance, indicating that the strength of functional pathways is also important for BCI performance, as is the modulation of these pathways.

The resting-state network (RSN) is thought to reflect the fundamental connectivity of the brain and thereby the amount of information that can be processed during mental tasks (Lee et al., 2020). Therefore, a person with an efficient RSN may have a better ability to perform motor imagery tasks (Zhang et al., 2015). Saha and Baumert (2020) stated that the resting-state network represents large scale spatiotemporal structures that underlie the baseline activity of a user and thereby variety in RSN can have implications on the user’s BCI performance. Sannelli et al. (2019) showed that varying peak amplitudes during Rest may indicate performance in MI-BCI tasks. However, in our study, no effect of such a resting state was observed. This might be due to the different definitions of resting-state in our study.

While past studies mostly defined resting-state as a complete absence of task, where the subject sits with closed eyes, we calculated resting-state from the first 3 s of each MI-BCI trial, where the subject looked at a fixation cross. This is different from past studies in that resting-states were shorter in duration and the subject’s eyes were open. Additionally, the subject could have been engaged in motor preparation, although the directionality of the MI trial (left or right hand) was only revealed after the cue appeared on the screen (see Figure 1). Thus, this motor preparation could not have impacted connectivity in laterality and might be better explained as motor readiness (Vidaurre et al., 2020). Nevertheless, the RSN data collected might reflect more connectivity than typical resting-state analyses by the nature of this motor preparation.

Interestingly, Vidaurre et al. (2020) also employed the pre-MI interval as a resting-state and compared it to the performance in the calibration run (where the subject has not yet received feedback). These results cannot be equally compared as the presence of feedback in our study could have a different impact on the resulting brain activity during motor imagery. And also during rest, the feedback presentation from the previous trial might have had a carry-over effect to the resting-state of the next trial (Carabalona and Castiglioni, 2009); for example, motivation is impacted by feedback (Alimardani et al., 2014; Pillette et al., 2021). Despite the inter-trial interval that was randomized between 0.5 and 2.5 s, this effect might still be prevalent.

### The Difference Between Task and Resting-State Connectivity

To investigate whether the difference in connectivity during motor imagery originated from a higher activation during MI or a lower activation during Rest, we evaluated the difference between MI and Rest. This approach was suggested by Gonuguntla et al. (2016), who showed that PLV increased during MI compared to Rest (Hamedi et al., 2016). Additionally, Li et al. (2019b) found that connections between motor areas in the brain increased during motor imagery, while the activity of the default mode network was suppressed. Therefore, we extracted the metric ΔPLV = PLV_MI_ − PLV_Rest_: when the value is positive, connectivity in MI is greater than during Rest. As successful motor imagery classification depends on the difference between those states, this indication might provide additional insights into the brain activity underlying MI. The results showed that in both hemispheres, the difference between MI and Rest is mostly negative for both efficient and inefficient users. This indicates that connectivity during MI is lower than connectivity during Rest, which is the opposite of what was expected following previous studies (Gonuguntla et al., 2016; Li et al., 2019a). Our results showed no significant difference between High and Low aptitude users on the connectivity change from Rest to the MI task. Further research is required to uncover the true dynamics of brain networks especially when BCI users transition between Rest and MI states.

### Global, Large and Local Scale Connectivity

Following previous studies, three different connectivity scales were evaluated in this study: the average connectivity over all the sensorimotor areas (e.g., Zhang et al., 2015), the inter-hemispheric connectivity between the left and right hemispheres, and the intra-hemispheric connectivity within each of the right and left hemispheres (e.g., Wang et al., 2006; Zhang et al., 2014; Vidaurre et al., 2020).

Significant results were only found in the local connectivity in the right hemisphere whereas previous literature pointed out the contribution of both hemispheres to connectivity during MI. For instance, Wang et al. (2006) studied six BCI users and showed that PLV connectivity of C3 and its surrounding electrodes (left hemisphere) was larger in right-hand MI, while for C4 and its surrounding electrodes (right hemisphere) it was *vice versa*; the connectivity was larger during left-hand MI. However, Zhang et al. (2014) reported the opposite; alpha band connectivity within the left hemisphere was greater in the left-hand trials while right hemisphere connectivity in the same alpha band was higher in the right-hand trials. The authors attributed this difference to higher mu rhythm desynchronization in the contralateral hemispheres during MI of one hand, which then resulted in a larger synchronization value in the ipsilateral areas. Thus, literature was not consistent regarding intra-hemispheric connectivity and its association with MI hand. While our results indicated a difference of alpha band connectivity between High and Low aptitude groups in the right hemisphere, we observed no further difference between the right and left MI trials (no significant effect for RL factor in Table 2).

Our finding of a larger right hemisphere connectivity in the High aptitude group can be discussed in the context of subjects’ handedness and its effect on SMR lateralization (Zapała et al., 2020). In our study, subjects were selected to be right-handed; Zapała et al. (2020) reported that right-handers are better MI-BCI performers and that they display greater alpha band desynchronization during left-hand MI than left-handers. They argued that a higher activation level in the right sensorimotor cortex of right-handers and consequently the distinction of ERD patterns between the two MI sides would lead to an effective BCI control in this group (Zapała et al., 2020). Our result further confirms this argument by showing that among right-handers, those who demonstrate larger connectivity in the right hemisphere are more efficient in MI-BCI control. This role of the right hemisphere can be perhaps related to the user’s ability to successfully engage in the non-dominant hand imagery. A recent case study focusing on twins with discordant dominant hands showed that the twin who was capable of producing more pronounced ERD during MI of the non-dominant hand obtained significantly higher BCI performance (Carino-Escobar et al., 2020). Therefore, future research should further investigate the role of the non-dominant hand and corresponding hemispheric connectivity on MI-BCI performance.

The fact that unlike previous studies no effects were found for the larger scales can be explained by various methodologies employed by these studies. For example, Wang et al. (2006) defined large scale connectivity by focusing on three channels of C3, C4, and FCz instead of the connectivity between all electrodes in the left and right hemispheres. Vidaurre et al. (2020) employed source localization before extracting the coherence values and in addition incorporated three classes in the MI task where foot imagery was also classified. The connectivity between hemispheres was significant in Zhang et al. (2014) only in the beta band; left to right connectivity was higher in left trials, while the right to left connectivity was higher in right trials. Thus, future research should attempt to bring more consistency to their methodology, in order to reveal the true underlying effects across studies.

### Limitations

The fact that our results could not fully replicate previous findings in the literature can be attributed to the practical limitations that existed in this study. As discussed in Leeuwis et al. (2021a), the sample was recruited using convenience sampling. This introduced a skewed distribution in gender and age of the subjects, which are both factors that might impact BCI performance (Randolph et al., 2010; Cantillo-Negrete et al., 2014). In addition, as we noted before, the sample was composed of only right-handed subjects. While this was necessary for controlling the number of factors and interpretation of the results for the majority of BCI users, it indeed limited our understanding of the dominant hemispheric connectivity and its role on BCI efficiency. Previous research has already indicated different inter-hemispheric and intra-hemispheric connectivity between right- and left-handers (Vukelić et al., 2019). Additionally, Zapała et al. (2020) suggested that left-handers show lower BCI accuracies due to weaker SMR modulation compared to right-handers and that right-handers demonstrate greater desynchronization patterns during left-hand imagery than right-hand. The latter might have induced effects of trial directionality on hemispheric activity due to non-randomized handedness. Thus, future research should look into the difference between right and left-handers regarding connectivity during motor imagery in order to increase the usability of MI-BCI across a broader range of possible users.

A known and unavoidable problem within connectivity analysis is the sensitivity to volume conduction or source leakage (Bruña et al., 2018; He et al., 2019). Connectivity measures might represent spurious correlations because EEG signals get spatially diffused between the source regions and the electrodes and thereby the recorded EEG signals may represent the activity of multiple active sources (Hamedi et al., 2016). The issue of volume conduction can be handled by using phase-lag models, which directly estimate functional connectivity between two regions. Although Phase-lag index (PLI) is considered a robust measure of volume conduction when compared to the PLV, it still possesses a serious drawback when it comes to the non-stationarity of EEG data (Tognoli and Kelso, 2020) as it is non-normalized and therefore still biased (Ewald et al., 2012). Originally, PLI was developed to estimate phase connectivity ignoring the contribution of zero lag (Stam et al., 2007), but the metric has shown a low test-retest reliability (Colclough et al., 2016). Furthermore, Cohen (2015) argues that the choice of a phase-based functional connectivity measure should depend on hypothesis-driven vs. exploratory analysis. While PLI reduces Type-I errors (i.e., false identification of connections) due to its insensitivity to volume conduction, it increases Type-II errors, which can lead to rejection of the null hypothesis when it actually should not (in other words, missing true connections). On the contrary, PLV reduces the risk of Type-II errors but is more likely to increase the Type-I errors due to its robustness to non-stationary data. Hence, PLI is preferred in exploratory analysis, whereas PLV is more suited to hypothesis-driven studies as well as studies with an interest in the time course of changes in connectivity (Cohen, 2015).

Given the hypothesis-based characteristics of this study, we opted for phase synchronization analysis using PLV, which does not involve the magnitude of the signals but rather the phase of the signals (Sakkalis, 2011). Additionally, in order to reduce the susceptibility of our analysis to volume conduction artifacts, we applied a Laplacian spatial filter to the EEG recordings (Cohen, 2014; Kayser and Tenke, 2015; Caicedo-Acosta et al., 2021). The problem of volume conduction, however, is still prevalent in PLV analyses and further refinement of phase synchrony estimation algorithms that are cost-effective and robust to volume conduction remains necessary (Bruña et al., 2018).

Previous studies have examined correlations between inferred brain activity obtained by localization algorithms (e.g., Lee et al., 2020; Vidaurre et al., 2020). Source localization aims to solve the inverse problem, where surface EEG recordings are translated to underlying brain structures while accounting for field propagation (He et al., 2019). While this allows for interpretation of the brain structures that underlie functional connectivity during MI, potential confounding effects might result from the employed reconstruction algorithms (Westlake and Nagarajan, 2011). As MI-BCI systems mostly rely on EEG measurements, the functional connectivity expressed between electrodes, as opposed to connectivity between brain structures, communicates interpretable values that future MI-BCI researchers can implement easily. Thereby, differences in connectivity, without the need for prior reverse engineering of the source location, can be employed as future features in determining High and Low aptitude users in MI-BCI experiments.

As noted, intra- and inter-subject variability is a problem within EEG measurements (Saha and Baumert, 2020). Additionally, the exact definition of frequency band varies among subjects (Haegens et al., 2014), as does the cortical regions in which MI-related activations occur (Hamedi et al., 2016). Due to specific imagery strategies of the subject, variability occurs between subjects and over time (Seghier and Price, 2018). Not every subject starts motor imagery immediately after the cue. This might also be reflected in connectivity. To adjust for these variations, Wang et al. (2019) already showed that choosing the optimal time window for each subject individually can improve their accuracy. Future research might identify the individual settings that explain the underlying connectivity dynamics and incorporate them for MI-BCI classification.

### Future Research

Future research could explore multiple connectivity measures in a larger sample, e.g., comparing the classification of effective and functional connectivity within the sample. Especially, recording more sessions in a longitudinal experiment will provide a more solid basis for establishing inefficient users and the relation between their BCI performance and connectivity values.

Additionally, the combination of neurophysiological characteristics, combined with users’ psychological and cognitive factors might further improve the identification of inefficient users (Leeuwis et al., 2021a). Within this dataset, future research could relate the factors identified in Leeuwis et al. (2021a), with the neurophysiological characteristics such as functional connectivity described in this study. Creating a more complete user profile can help to identify inefficient users and adapt training settings beforehand.

Moreover, as Zhang et al. (2020) suggested, categorizing inefficient users based on their resting-state EEG activity or offline/online BCI performance in order to provide targeted solutions for each group (e.g., employ new EEG feature, apply transfer learning algorithms, or develop new training strategies and experimental paradigms), can reduce the BCI inefficiency problem in a more effective way and speed up BCI mainstream adoption. Our study particularly highlights the potential of EEG functional connectivity in identifying inefficient users early on in research. Further studies are required to confirm the efficacy of this metric as a reliable classification feature for MI-BCI systems. Employing a large sample of inefficient users in order to test a connectivity-based BCI classifier might sound futuristic as long as the calculation is taking too much time (Zhang et al., 2014) but with the increasing computing power and better knowledge of the key factors, this gradually becomes a more realistic experimental set-up. Thus, with these expanding possibilities, future research should aim at identifying online classification accuracy “boosters”, such as functional connectivity, in order to solve MI-BCI inefficiency.

As discussed in Vidaurre et al. (2020), motor imagery is originating from somatosensory and motor cortices and is therefore related to a feeling of agency as well as the proprioceptive sensations the users’ experiences (Nikulin et al., 2008). Several studies showed that this proprioception after MI is correlated to increased performance (e.g., Ramos-Murguialday and Birbaumer, 2015; Vidaurre et al., 2019). The coexistence of motor initiation and the anticipation of the effects of the movements explains why connectivity between motor and sensory cortical areas relates to successful motor imagery. Making connectivity part of the online feedback feature set thereby makes sense. The use of this has already been shown to enhance BCI classification (Zhang et al., 2019; Gu et al., 2020) and might therefore be explored in future research.

Furthermore, classification scores of low performing users can be improved by incorporating new AI algorithms such as deep learning methods on raw EEG signals instead of the classical machine learning approach that relies on EEG feature extraction (e.g., Stieger et al., 2021; Tibrewal et al., 2021; Zhang et al., 2021). Deep learning models have the advantage of facilitating end-to-end learning; they can exploit information from raw data on their own, which is not only computationally more effective but also captures brain activity patterns underlying MI beyond the defined ERD features (Tibrewal et al., 2021). Particularly, in the light of connectivity research, deep learning provides a more holistic analysis of brain activity patterns during MI that extends beyond mu suppression in the sensorimotor area and this can result in a better discriminative power of BCI classifier for the inefficient users.

Last but not least, several studies point to the improvement of feedback and training methods through immersive and engaging environments, such as robotic platforms (Alimardani et al., 2018), virtual reality (Coogan and He, 2018), and gamification (de Castro-Cros et al., 2020). This resonates well with past research on user motivation and its effect on BCI performance (Nijboer et al., 2010; Alimardani et al., 2014; Sannelli et al., 2019; Kleih-Dahms et al., 2021). Therefore, employing state-of-the-art classification methods as well as training/feedback design is a promising avenue for future research to reduce the prevalence and severity of the BCI inefficiency problem.

## Conclusion

In this study, we examined the difference between high and low aptitude motor imagery BCI users in their EEG functional connectivity in three network scales (Global, Large, and Local scale) during the resting state, motor imagery task, and the transition between the two phases in each trial. Our comparison of two groups of High and Low BCI performers (each 27 subjects) showed that alpha-band functional connectivity in the right hemisphere was significantly higher in High aptitude MI-BCI users when they performed the motor imagery task. However, connectivity during resting-state and other scales were not found to be significantly different between High and Low MI-BCI performers. These findings add to the existing literature by providing a comprehensive analysis of functional connectivity at different network scales and during different phases of the MI task using a large sample of subjects. Our results confirm that indeed connectivity might be a valuable feature in user profiling for BCI experiments. However, this is not yet the end; to solve the MI-BCI inefficiency problem, future research should confirm the efficacy of functional connectivity as an online classification feature in a state-of-the-art MI-BCI paradigm with a large sample size. This verification will establish whether functional connectivity is truly able to distinguish motor imagery patterns and improve accuracy for users that are inefficient with current models.

## Data Availability Statement

The original contributions presented in the study are included in the article/Supplementary Materials, further inquiries can be directed to the corresponding author.

## Ethics Statement

The studies involving human participants were reviewed and approved by Research Ethics Committee of Tilburg School of Humanities and Digital Sciences (REDC #20201003). The patients/participants provided their written informed consent to participate in this study.

## Author Contributions

NL and MA designed the research. NL conducted the experiment. SY conducted the connectivity calculation and statistical analysis report, both under supervision of MA. NL wrote the manuscript with input from MA and SY. All authors contributed to the article and approved the submitted version.

## Conflict of Interest

The authors declare that the research was conducted in the absence of any commercial or financial relationships that could be construed as a potential conflict of interest.

## Publisher’s Note

All claims expressed in this article are solely those of the authors and do not necessarily represent those of their affiliated organizations, or those of the publisher, the editors and the reviewers. Any product that may be evaluated in this article, or claim that may be made by its manufacturer, is not guaranteed or endorsed by the publisher.
